# Do arrestin oligomers have specific functions?

**DOI:** 10.46439/signaling.1.009

**Published:** 2023

**Authors:** Vsevolod V. Gurevich

**Affiliations:** 1 Department of Pharmacology, Vanderbilt University, Nashville, TN 27232, USA

**Keywords:** Arrestin, GPCR, Cell signaling, Oligomers

## Abstract

Arrestins are a small family of versatile regulators of cell signaling. Arrestins regulate signaling and trafficking of G protein-coupled receptors, regulate and direct to particular subcellular compartments numerous protein kinases, ubiquitin ligases, etc. Three out of four arrestin subtypes expressed in vertebrates self-associate, each forming oligomers of a distinct size and shape. While the structures of the solution oligomers of arrestin-1, −2, and −3 have been elucidated, no function specific for the oligomeric form of either of these three subtypes has been identified thus far. Considering how multi-functional average-sized (~45 kDa) arrestin proteins were found to be, it appears likely that certain functions are predominantly or exclusively fulfilled by monomeric and oligomeric forms of each subtype.

## Introduction

Arrestins were discovered as key players in the conserved two-step homologous desensitization of G protein-coupled receptors (GPCRs): they specifically bind active phosphorylated GPCRs, precluding their coupling to cognate G proteins, thereby stopping (“arresting”) G protein-mediated receptor signaling [1]. Vertebrates have four arrestin subtypes, two specialized visual and two nonvisual [2]. Visual arestin-1^a^ is expressed at very high levels in rod [3,4] and cone [5] photoreceptors of the retina. Arrestin-4 is expressed at much lower level and only in cones [5]. Non-visual arrestin-2 and −3 are expressed in virtually every cell in the body, with most cells expressing a lot more arrestin-2 than arrestin-3 [6,7]. The primitive chordate lancelet, as well as the closest relatives of chordates, ascidians, express only one arrestin subtype. Thus, it is likely that the four arrestin genes in vertebrates emerged as the result of two rounds of whole genome duplication [2]. This idea is supported by the fact that bony fish that underwent a third round of whole genome duplication express seven arrestin subtypes (out of theoretically possible eight) [2]. GPCRs regulated by arrestins play key role in our vision, senses of smell and taste, and respond to various hormones, neurotransmitters, and other biologically active molecules. Mutations in different GPCRs underlie numerous congenital disorders ([8,9] and references therein). Loss-of-function mutations in arrestin-1 result in night blindness [10,11]. Three out of four vertebrate subtypes, arrestin-1, −2, and −3, self-associate, each forming a different type of oligomer.

## Arrestin-1

Bovine, mouse, and human arrestin-1 forms dimers and tetramers [12]. Although dimerization and tetramerization constants significantly differ between these mammalian species [12], the structure of arrestin-1 oligomers in solution (which was carefully elucidated for the bovine arrestin-1 [13, 14]) appears to be conserved, as homologous mutations in bovine and mouse proteins similarly suppress self-association [12].

The first discovered function of arrestin-1 was quenching rhodopsin signaling: it binds light-activated phosphorylated rhodopsin and precludes its coupling to the cognate heterotrimeric G protein, transducin [15,16]. It has been demonstrated that only monomeric arrestin-1 can bind rhodopsin *in vitro* [13]. Indeed, oligomerization-deficient mouse arrestin-1 mutant transgenically expressed in rods of arrestin-1 knockout mice quenched rhodopsin signaling *in vivo* pretty much like the wild type protein [17]. Arrestin-1 was found to bind microtubules in cells at concentrations where it was predominantly monomeric [18]. However, the binding to polymerized tubulin, in sharp contrast to its binding to rhodopsin, did not cause the dissociation of arrestin-1 oligomers *in vitro* [13], suggesting that oligomeric arrestin-1 also binds microtubules. The interaction of one molecule of arrestin-1 with one molecule of rhodopsin was demonstrated in living mice [4], with purified proteins *in vitro* [4,19], and later confirmed by the crystal structure of the complex [20,21].

All these findings are consistent with the idea that arrestin-1 oligomers are the storage form, whereas the monomer is an active form [22]. The finding that the expression of oligomerization-deficient arrestin-1 causes light-independent photoreceptor death *in vivo* [17] at levels where the wild type protein is harmless [3] indicates that the monomer does something bad, which the oligomers do not do. This suggests that arrestin-1 oligomerization is cytoprotective: rods need to express arrestin-1 at levels comparable to those of rhodopsin, and its self-association prevents the monomer concentration from reaching toxic levels. The molecular mechanism(s) underlying cytotoxicity of monomeric arrestin-1 remain unknown.

Thus, at the moment the only thing we know that arrestin-1 oligomers actually do is bind microtubules [13]. This might increase their capacity for binding arrestin-1, as microtubules serve as the “parking place” of this extremely abundant protein away from rhodopsin-containing rod outer segments in the dark, when its rhodopsin-quenching function is not needed [23]. However, this does not seem satisfying: as a rule, everything that exists in living cells has a function. Arrestin-1 certainly has more binding partners than just rhodopsin and microtubules. Its interaction with clathrin adaptor AP2 [24] appears to depend on rhodopsin binding-induced release of its C-terminus, where the AP2 binding site is located. Thus, it is likely also a function of the monomer. It remains to be elucidated whether monomeric or oligomeric form of arrestin-1 interacts with other known binding partners, NSF [25] and enolase-1 [26]. It is likely that more arrestin-1 binding partners will be discovered, some of which might prefer arrestin-1 oligomers.

## Arrestin-2

Arrestin-2 was cloned by homology with arrestin-1 [27] and originally named β-arrestin, because it showed preference for β_2_-adrenergic receptors over rhodopsin, in sharp contrast to arrestin-1 that demonstrated the opposite preference [28]. Subsequent studies demonstrated that this arrestin subtype readily interacts not only with β_2_-adrenergic receptors, but with numerous non-visual GPCRs [29]. Therefore, here we use systematic names of arrestin proteins, where the number after the dash indicates the order of cloning and does not imply anything else. The preference of arrestin-1 for rhodopsin and of arrestin-2 for non-visual receptors was demonstrated in experiments with arrestin-1/2 chimeras, which identified two homologous elements in both subtypes responsible for their receptor specificity [30,31].

An abundant intracellular metabolite inositol-hexakisphosphate (IP_6_) inhibits the oligomerization of arrestin-1 but facilitates the oligomerization of arrestin-2 [32]. Originally the self-association of arrestin-2 was analyzed using the same monomer-dimer-tetramer model that worked well for arrestin-1 [32]. However, at higher concentrations arrestin-2 was shown to form oligomers greater than the tetramer, suggesting that a different model would be more appropriate [33]. Further studies revealed that in the presence of IP_6_ in solution arrestin-2 forms chains with no apparent limits, where all molecules appear to be in the basal conformation [33]. The chains are likely similar to those observed in arrestin-2 crystals soaked with IP_6_ [34]. In these chains identified receptor-binding elements (reviewed in [35,36]) of every arrestin-2 molecule are shielded by sister protomers, suggesting that only the monomeric form can bind GPCRs. Indeed, in all solved structures of the complexes of arrestin-2 with various receptors arrestin-2 monomer was found bound to a single GPCR molecule [37-42]. Analysis of the arrestin-2 interactions with several receptors in living cells using cross-linking via unnatural amino acids suggested exactly the same arrangement [43,44].

In cells arrestin-2 is localized to both the cytoplasm and nucleus [45]. Cell culture studies suggest that while the monomer can enter the nucleus, the oligomeric arrestin-2 cannot and therefore remains in the cytoplasm [34,46]. Thus, it was hypothesized that oligomerization serves to prevent arrestin-2 from entering the nucleus. No other function of arrestin-2 oligomer was suggested so far.

## Arrestin-3

Arrestin-3 is the second vertebrate non-visual subtype [47,48]. It appears to be even more promiscuous in terms of receptor specificity than arrestin-2 [49], possibly due to a more lose structure of its C-domain [50]. In contrast to highly homologous arrestin-2 [47,48], in the presence of IP_6_ arrestin-3 forms trimers, the structure of which was recently solved [51]. Importantly, in these trimers each protomer of arrestin-3 assumes receptor-bound-like conformation with characteristic twist of the two domains relative to each other [51]. Thus, in contrast to arrestin-1 and −2, oligomerization of arrestin-3 involves a conformational rearrangement. Based on this finding, IP_6_ was proposed to serve as an activator of arrestin-3, similar to GPCRs [52]. It was hypothesized that its activation by IP_6_ is the mechanistic basis of receptor-independent activation of JNK family kinases by arrestin-3 [52]. However, two lines of experimental evidence contradict this hypothesis. First, functional analysis of a large set of mutants showed that the ability of arrestin-3 to bind receptors (i.e., to assume receptor-bound conformation) and to facilitate JNK3 activation have different, essentially opposite, structural requirements [53]. Second, short arrestin-3-derived peptides lacking most elements involved in both receptor binding and trimerization, were found to efficiently facilitate the activation of JNK3 in cells [54,55]. Similar to arrestin-2, the oligomerization of arrestin-3 was shown to prevent it from entering the nucleus [46]. However, monomeric arrestin-3 is almost exclusively cytoplasmic due to the presence of an efficient nuclear export signal in its C-terminus [45]. Thus, in contrast to arrestin-2, the oligomerization of arrestin-3 does not change its subcellular localization. So far, no specific function of the oligomeric form of arrestin-3 has been described.

## Unanswered Questions

According to in-cell data, arrestin-2 and −3 from hetero-oligomers [46]. As the shape of the oligomeric forms of these two proteins is dramatically different [33], it is unclear what shape their hetero-oligomers have. This needs to be determined experimentally.

Arrestin-2 and arrestin-3 are encoded by multi-exon genes [2]. Not surprisingly, both non-visual arrestins have splice variants [48]. The prevalent form of arrestin-2 has an eight-residue insertion in one of the loops encoded by a separate exon that the prevalent form of arrestin-3 does not have. The structure of the arrestin-3 trimer [51] shows that if these extra residues were present, the extended loop would clash with sister protomers, precluding trimerization [33]. This was confirmed experimentally: arrestin-3 with the insertion of arrestin-2-specific eight residues does not form trimers [33]. Arrestin-2 has a splice variant lacking these eight residues [48]. It remains to be elucidated whether this variant forms chains, like the predominant splice variant of arrestin-2, or can trimerize with the change of conformation, like arrestin-3.

The most important question is whether oligomers of arrestin-1, −2, and −3 have specific functions, i.e., can do something that corresponding monomers cannot. Arrestins are average-sized ~45 kDa proteins. Non-visual arrestins were shown to have numerous interaction partners and fulfil many functions in cells (reviewed in [35,36,56]). It was hypothesized that the conformational flexibility of arrestins explains how these relatively small proteins can do so many different things [57-59]. However, arrestins in the cytoplasm exist in at least four distinct molecular forms: free and microtubule-bound monomers and oligomers. It is entirely possible that some of these functions are specific for the particular forms of arrestins. Construction of oligomerization-deficient arrestin-2 and −3, as well as mutants that do not bind microtubules, and comparison of their functional capabilities with those of wild type proteins appears to be the most straightforward way to determine whether this is the case.

Aberrant cellular signaling underlies virtually all human disorders, with the exception of infectious diseases. Understanding the molecular mechanisms of cell signaling, many branches of which are affected by arrestin proteins, is necessary for the development of effective therapies.

## Footnotes

a We use systematic names of arrestin proteins, where the number after the dash indicates the order of cloning: arrestin-1 (historic names S-antigen, 48 kDa protein, visual or rod arrestin), arrestin-2 (β-arrestin or β-arrestin1), arrestin-3 (β-arrestin2 or hTHY-ARRX), and arrestin-4 (cone or X-arrestin).
